# Beyond the parish pump: what next for public health?

**DOI:** 10.1186/s12889-018-5791-0

**Published:** 2018-07-11

**Authors:** Alex Hall, Jonathan Hammond, Donna Bramwell, Anna Coleman, Lynsey Warwick-Giles, Kath Checkland

**Affiliations:** 1 Division of Population Health, Health Services Research & Primary Care, School of Health Sciences, University of Manchester, Manchester, UK; 2 Division of Nursing, Midwifery & Social Work, School of Health Sciences, University of Manchester, Manchester, UK; 3 Manchester Academic Health Science Centre, Manchester, UK

**Keywords:** UK, Health policy, NHS, Organising healthcare, Population health, Professionalism, Professional identity, Public health

## Abstract

**Background:**

Public health has had a history characterised by uncertainty of purpose, locus of control, and workforce identity. In many health systems, the public health function is fragmented, isolated and under-resourced. We use the most recent major reforms to the English National Health Service and local government, the Health and Social Care Act 2012 (HSCA12), as a lens through which to explore the changing nature of public health professionalism.

**Methods:**

This paper is based upon a 3-year longitudinal study into the impacts of the HSCA12 upon the commissioning system in England, in which we conducted 141 interviews with 118 commissioners and senior staff from a variety of health service commissioner and provider organisations, local government, and the third sector. For the present paper, we developed a subset of data relevant to public health, and analysed it using a framework derived from the literature on public health professionalism, exploring themes identified from relevant policy documents and research.

**Results:**

The move of public health responsibilities into local government introduced an element of politicisation which challenged public health professional autonomy. There were mixed feelings about the status of public health as a specialist profession. The creation of a national public health organisation helped raise the profile of profession, but there were concerns about clarity of responsibilities, accountability, and upholding ‘pure’ public health professional values. There was confusion about the remit of other organisations in relation to public health.

**Conclusions:**

Where public health professionals sit in a health system in absolute terms is less important than their ability to develop relationships, negotiate their roles, and provide expert public health influence across that system. A conflation between ‘population health’ and ‘public health’ fosters unrealistic expectations of the profession. Public health may be best placed to provide leadership for other stakeholders and professional groups working towards improving health outcomes of their defined populations, but there remains a need to clarify the role(s) that public health as a specialist profession has to play in helping to fulfil population health goals.

## Background

In 1854, a London physician, John Snow, persuaded the parish authorities in Soho to remove the handle of the water pump that his statistical analysis suggested was responsible for spreading cholera amongst the population. This simple action highlights the important role of public health professionals in the era when infectious diseases were the prime cause of premature death. Meticulous epidemiological research, followed by simple and direct action, saved lives. However, since that time, and most particularly since the Second World War, public health has had a history characterised by uncertainty of purpose, locus of control, and workforce identity [1]. In the UK, repeated reforms of the National Health Service (NHS) and local government since 1974 have been particularly unsettling for the field [2], but similar difficulties are present in other jurisdictions. A report from the World Health Organization Regional Office for Europe [3] argues that:

*In many health systems, the public health function is fragmented and sections of the workforce may feel isolated. There are often continuing problems of under-resourcing, skill shortages, insufficient capacity, poor morale and low pay. In order to meet population health needs, significant efforts are required to scale up not only the number of public health professionals, but also their quality and relevance to public health.* (p.17).

The WHO’s solution is to call for European-wide action to strengthen public health capabilities, identifying ten areas of work (Table 1):

**Table 1:** Ten Essential Public Health Operations [3]

1. Surveillance of population health and well-being	
2. Monitoring and response to health hazards and emergencies	
3. Health protection, including environmental, occupational, food safety and others	
4. Health promotion, including action to address social determinants and health inequity	
5. Disease prevention, including early detection of illness	
6. Assuring governance for health and well-being	
7. Assuring a sufficient and competent public health workforce	
8. Assuring sustainable organizational structures and financing	
9. Advocacy, communication and social mobilization for health	
10. Advancing public health research to inform policy and practice	

The report identifies three categories of workers with responsibility for public health: public health specialists; all health professionals; and other workers whose work may impact upon the health of the public, including those involved in housing, transport and education [3]. Similar lists of essential operations and stakeholders exist for the public health system in the USA [4]. Comprehensive lists of essential activities, coupled with very wide definitions of the ‘public health workforce’, raise some issues for public health professionals, particularly in relation to professional identity, areas of jurisdiction and relationship to other professions. These challenges may be especially relevant to public health’s role in strengthening health systems. This can be particularly difficult in international work within developing countries that struggle to finance population-scale healthcare, and may be resistant to Western-centric delivery of interventions that is ignorant of their complex socio-political contexts [5, 6].

In this paper we use the most recent reorganisation of the NHS in England as a lens through which to explore these issues. A centre piece of the reforms, enacted in the Health and Social Care Act 2012 [7] (hereafter ‘HSCA12’ or ‘the Act’), was the shift of responsibility for public health from the NHS to local government authorities (LAs), and the creation of a new national body, Public Health England (PHE), with responsibility for unifying the profession and providing support for those delivering public health services [8]. We report findings from a study designed to explore the impact of the HSCA12 on local health economies, using theoretical understandings of professional identify and professionalism to explore the public health profession in the new system. Setting current issues in a historical context, we use our findings to reflect more generally on the roles and professional identities of public health professionals in a complex world, and to offer some tentative conclusions about future avenues for the development of public health professionalism.

### Theoretical framework: Professional identity and professionalism

To identify as a professional is to embody the characteristics, values, obligations and norms of that profession [9]. Today, public health is commonly defined as *“the science and art of preventing disease, prolonging life and promoting, protecting and improving health through the organised efforts of society”* [10]. Despite the very broad definitions of public health and of the public health workforce, it can be argued that to identify as a public health professional is to embody the emphasis upon collective responsibility and partnership working to improve population health through the three core practice domains of health protection, health improvement, and health service improvement [11]. Additionally, public health is informed by a history of standing up for vulnerable populations; for public health professionals, *“shying away from advocacy is comparable to medical negligence”* [12].

Professional identification is underpinned by professionalism, a complex concept that may be divided into three dimensions: intrapersonal (behaviours, traits); interpersonal (relationships, actions) and societal (wider responsibilities) [13]. It is a ‘social contract’ between professionals and society, granting professionals permission to use their expertise in the understanding that they will provide certain services and adopt certain behaviours [14]. Perspectives upon professionalism have changed over time. The most firmly-established professional group in healthcare, the medical profession, has moved from enjoying an unconditional position of privilege and respect to enduring increasing scrutiny of its specialist status [15]. Today, healthcare work is often carried out by interprofessional, multi-disciplinary teams rather than individuals, controlled and monitored by mechanisms inherent to the evidence-based practice paradigm [15], serving patients who are cast as active informed consumers rather than passive recipients of paternalism [16]. These changes illuminate multiple perspectives upon professionalism: traditional ‘pure’ forms that promote occupational discretion and self-regulation; ‘controlled’ forms that emphasise managerialism and standardisation; and ‘hybrid’ forms that adopt aspects of both of these seemingly contradictory perspectives in the renegotiation of professional identities [17].

Noordegraaf argues that hybrid forms of professionalism are moving beyond an initially awkward marriage between pure and controlled perspectives, to more legitimate ‘organising’ forms in which professionals see organising work as an intrinsic part of their professional identity [17]. McGivern et al. suggest that professionals need to learn how to embody hybridity as part of their identities, rather than seeing management as a reluctant appendix. Learning to modify and adapt elements of the professional role, or what it constitutes and means to be a professional, is driven by the nature of the institutional environment [18]. Professional roles are hybridised through the need to meet perpetually changing organisational expectations, adapting, co-ordinating and mediating to reconcile the competing ideologies and interests of their own professional identity and those of the organisation [19].

To provide contextual background pre-HSCA12, we now turn to consider how pure, controlled, hybrid and organising forms of professionalism have manifested themselves in public health through the history of the profession. We then present the methods used in our longitudinal study of the influence of the HSCA12 upon the commissioning system, and use this theoretical framework of professionalism to explore the specific impacts of the Act upon public health professionalism.

### A short history of professionalism in public health in the UK

#### 1840–2010

Public health emerged in the context of the industrial revolution and an associated need to address sanitation and infection transmission [20]. Responsibility for public health was initially given to LA Medical Officers of Health (MOsH), and during the nineteenth and early twentieth centuries, public health developed a form of ‘pure’ professionalism that foregrounded an epidemiological and laboratory-focused approach, applied in conjunction with other established occupational groups including sanitary engineers and town planners [21]. The creation of the NHS in 1948 introduced a tripartite structure of hospitals, primary care, and LA health services, dominated by hospitals, that led to a gradual erosion of LA responsibility for public health over the 1950s and 1960s [22]. During this time, the distinct form of professionalism for public health that had been developing since the mid-nineteenth century was challenged by an increasing medicalisation, culminating in the watershed 1974 reforms to NHS and local government.

The 1974 reforms saw many public health functions transferred into the NHS, with public health redefined as the speciality of ‘community medicine’, and MOsH become NHS ‘community physicians’ [1]. The Faculty of Community Medicine, formed in 1972, mandated medical qualification as a membership criterion [2]. However, attempts to define public health as a medical subcategory were infused with confusion about the administration and accountability within community medicine, and the remit and necessary skills of the community physician [2, 23].

Internationally, the WHO began to develop a new philosophy of public health focussing on health promotion [24], which became established as a central pillar of the ‘new public health’ movement [20]. This movement was recognised in the UK [25], where the name ‘public health’ was reclaimed, and Directors of Public Health (DsPH) were established within the NHS [2]. It continued apace during the 1990s, particularly following the 1997 election of the New Labour government. Public health became increasingly multidisciplinary: the Health Protection Agency was formed to unite environmental, chemical, microbiology and epidemiology services [21]; DsPH were appointed jointly across the NHS and LAs [1]; and the Faculty of Public Health (formerly the Faculty of Community Medicine) relaxed its demand on mandatory medical qualification [2].

Despite this, emphasis upon medical leadership in public health endured [1]. Although New Labour policy rhetoric espoused new public health values, the NHS retained a focus on treating ill health rather than embracing health promotion [26], corresponding with the adoption of a business-like approach to administration (often called New Public Management) that emphasised targets as a governance mechanism, and the importance of marketisation of the NHS [27]. A complex hybrid form of professionalism thus emerged within public health, characterised by an uneasy combination of features associated with a ‘pure’ public health, undercut by an enduring emphasis upon medicalisation, and uncertainty associated with moves towards controlled professionalism in an era of increasing managerialism.

#### 2010 – Present: The health and social care act 2012

Following the 2010 UK general election, the new Coalition government published policy documents focusing upon the NHS and public health [28–30]. The main stated aims were to put clinicians at the heart of commissioning services, and to give them greater freedom to ensure that services met the needs of local populations. To achieve this, significant changes to structure and responsibilities were instituted. The managerially-led commissioning organisations, Primary Care Trusts (PCTs), were replaced with clinically-led Clinical Commissioning Groups (CCGs). Responsibility for system management and oversight was transferred from the Department of Health to a new Arms’ Length Body, NHS England (NHSE). Prior to the HSCA12, PCTs were responsible for commissioning all the services required by their local geographical populations, including primary, community, secondary and specialist services, as well as public health. The HSCA12 divided responsibilities for different types of service to different organisations: CCGs were given responsibility for commissioning community and secondary care services, whilst NHSE took responsibility for commissioning primary care and specialised services, and for overseeing CCGs. Commissioning Support Units were established to provide support to clinical commissioners, competing for CCG business in the internal marketplace of the NHS [31].

Responsibility for public health was transferred back to LAs, with the argument that local government would be a better setting than the NHS from which to influence the wider determinants of health [30], linking directly to services such as housing, education and leisure. LAs thus inherited responsibility for a range of public health services previously commissioned by PCTs, including obesity programmes, tobacco control, most sexual health services, and drug or alcohol misuse services [32]. DsPH were transposed from PCTs into senior roles within LAs, and supported by a ring-fenced public health budget [30]. NHSE was given responsibility for a small number of public health functions, notably national screening and immunisation programmes [33]. The reforms also saw the creation of Public Health England (PHE), an executive agency of the Department of Health, to support LAs and the NHS to deliver public health responsibilities and to unify the diverse world of public health specialists [8]. A new Public Health Outcomes Framework was developed to direct public health activity [34].

These changes were widely welcomed, but have raised a number of challenges. The organisation of public health teams has been characterised by instability, with local variations across LAs and within individual authorities that have continued to restructure and rearrange following the reforms [35]. There has been increased fragmentation, as responsibilities for different components of some types of care are now split between LAs, CCGs and providers; public health teams that previously worked together in PCTs have sometimes been separated out into these different organisations [36]. Budget cuts, said to undermine the focus upon longer-term prevention, are likely to be amplified by the removal of the ring-fencing of the public health budget from 2018 [37]. Public health teams have also had to adjust to very different working environments, including working alongside elected officials with diverse agendas. Research evidence often needs to be seen as commensurate with the LA agenda if it is to inform commissioning [38]. There are also questions over the levels of autonomy afforded to DsPH within LAs [35, 36], and they have to balance being independent experts who are also seen to be promoting their LA’s agenda [37].

As the national public health agency, PHE has operational autonomy to exercise professional judgment and advocate evidence-based practice [39]. However, as an executive agency it is closer to the Department of Health than was its predecessor Health Protection Agency, with employees bound by the civil service code of conduct, and there have been claims of political interference in the scope and focus of its work [12]. Some public health professionals have highlighted other challenges to the unifying agenda of PHE from the multiplicity of stakeholders inherent to a localism agenda [40], as well as from the fragmenting of responsibilities. Thus, for example, screening and immunisation programmes are commissioned by NHSE but supported by PHE teams embedded within NHSE regional teams, which in some cases have led to increased complexity and fragmentation of accountabilities [41].

The move of public health to LAs was welcomed by some as a ‘return home’ [22], implying in part a return to a familiar ‘pure’ professionalism. However, the creation of PHE within the civil service suggests elements of a more ‘controlled’ form of professionalism, with advocacy less important than managerialism and standardisation. The fragmentation that we have identified, with public health staff moving in complex ways between the different organisations, highlights how hybrid professional identities arise out of local and particular engagements between individuals and their organisations [19].

In this paper we use this recent reorganisation of the public health function in the English NHS as a lens through which to interrogate this local and situated negotiation of new hybrid professional identities within public health. We build upon the themes which have emerged in this brief historical analysis, exploring autonomy, local variation, the role of a national public health body, and wider perceptions of public health roles. Our discussion pulls these themes together to consider the implications for the WHO public health agenda.

## Methods

The work in this paper was developed iteratively during a large, longitudinal mixed methods study that explored the impacts of the HSCA12 upon the commissioning system in England, conducted between January 2015 and December 2017. Ethics approval was given by the University of Manchester research ethics committee in March 2015.

The study adopted a realist approach [42], focusing on a number of research questions developed to explore the complexity of the new system, how commissioning was being conducted, and changes to quality and outcomes. The HSCA12 was a far-reaching set of reforms affecting most aspects of the English health system, and the study was designed to allow exploration of the breadth and depth of these reforms. It included a number of complementary qualitative work streams, to explore:

- The operation of the new commissioning system;
- The roles of different stakeholders in the new system, through focus on specific ‘tracer’ areas of service commissioning: dementia services (to explore joint commissioning of health and social care); orthopaedics (to illuminate the role of CCGs); specialised services (to investigate the role of NHS England); sexual health and screening (to explore changes to public health commissioning);
- The views of stakeholders on what had been achieved 4 years after the reforms were enacted.

The study took place in two metropolitan areas of England, both with populations of over a million people. These areas corresponded to two regions within the English NHS structure that form distinct local health economies, each containing a large city. They were chosen as case studies because of their socioeconomic diversity, and because they were large enough to allow exploration of interactions between numerous commissioners and other stakeholder organisations.

Overall, we conducted 141 interviews with 118 commissioners and senior staff from CCGs, LAs, PHE, NHSE, Commissioning Support Units, provider organisations and the third sector (a minority of interviews contained more than one participant; some participants gave repeat interviews). We sampled participants purposively for variation in organisation type and personal role, including a snowball approach whereby participants recommended additional interviewees. Interviews focused upon continuities and changes within the commissioning system pre- and post-HSCA12, and included exploration of organisational and personal roles, key successes and challenges, decision-making and accountability, performance management, relationships, and communication between organisations and people. They were conducted either face-to-face at participants’ workplaces, or over the telephone, and all were audio-recorded. Participants were provided with written information about the study, and gave written consent, or for telephone interviews gave consent verbally at the beginning of the interview.

Data collection and analysis occurred simultaneously, so that emerging findings could inform subsequent interviews. This continued until thematic saturation, when no new findings emerged. All interviews were transcribed verbatim, and transcripts imported into NVivo 10 software. Analysis involved coding data extracts according to a frame developed from our understanding of the commissioning system and the HSCA12 policy documentation, complemented by emerging concepts. To help visualise the process of commissioning in each area, we produced A3-sized diagrams mapping interactions between organisations. Findings were regularly discussed at team meetings, with analytical memos recording conceptual development.

Our analysis of the impact of reforms on public health commissioning revealed strong views relating to the effect of the changes upon public health professionalism. These data were therefore further analysed to explore this concept in more depth, addressing the following research question: *how are local public health actors negotiating new professional identities following the introduction of the HSCA12 reforms?* It interrogated a relevant subset of data from the main study that captured participants’ talk about public health. This subset was developed from (a) the full transcripts of interviews with participants recruited because of their roles in public health, and (b) a search of all remaining transcripts for the words ‘public health’, with relevant surrounding text extracted. This subset incorporated data from 55 interviews conducted with 50 of the 118 participants in the main study. Further detail about these participants is provided in Table 2.

**Table 2:** Participants by organisation type and area

Organisation type	Area 1	Area 2	National level
Clinical Commissioning Group	9	11	
Local Authority	13	6	
NHS England and/or Public Health England	5	1	1
Commissioning Support Unit	1		
Other	1 (organisational consultant)		2 (screening advisor)

We reanalysed these data through the theoretical lens of professionalism outlined above, within a broad thematic framework identified from the relevant policy literature and nascent research into public health post-HSCA12: (1) autonomy within LAs; (2) local variations; (3) role of PHE; (4) other commissioners’ perspectives upon public health.

## Results

Results are presented according to the broad thematic framework above. Quotes presented are chosen because they are representative of a broad body of opinion within the dataset, or because they highlight specific issues associated with the new system which shed light upon the topic as a whole.

### Autonomy within local authorities

Participants reflected on the levels of autonomy that public health teams, particularly DsPH, were afforded in LAs in comparison to within PCTs of the pre-Act system. There were perceptions of a reduction in autonomy due to the DsPH now reporting to the ‘cabinet’ of elected councillors:

*in PCTs, I think Directors of Public Health… could make decisions quite easily… [now] the ultimate decision doesn’t lie with the Director of Public Health any more, it lies above that with cabinet.* [10,944, LA, Area 1, March 2016].

This curtailed autonomy emphasised the importance of upholding the advocacy principles at the heart of ‘pure’ public health professionalism, in particular, the need to stand up for services that might fall victim to politicisation in a climate of financial challenge:

*public health, sexual health spend might be under closer scrutiny… and I’d be concerned about those things being vulnerable. It’s then up to us to make the argument, and for the Director of Public Health to work with the powers that be to make sure they understand it and appreciate that it’s important.* [12649, LA, Area 2, May 2016]

This need for DsPH to be able to persuade decision-makers within a politicised environment seemed to be a new, post-Act feature of the hybridity of the public health professional role, which was not universally welcomed:

*as DPH… it was really difficult to actually see what I’d achieved… I just thought the people that were far better at all of the politicking and the relationship management I guess than I was, I preferred the hard science… so it just wasn’t a terribly natural fit.* [19974, former DPH (now PHE), Area 1, March 2016]

The attention to ‘politicking and relationship management’ that formed part of the new hybrid role within the LA appeared to be unsatisfying to some participants when compared to the more pure, technical side of public health work. However, other colleagues would more naturally embrace this aspect of the new hybrid role.

### Local variations

Conceptualisations of public health professionalism varied in different LAs across each of our geographical areas. In Area 2, one LA team seemed to prefer a more controlled, managerial form of professionalism through scrutiny of outcomes, whereas another LA team preferred a more hybridised form that emphasised interaction and co-operation with their PHE/NHSE colleagues. These differences in approach suggested that variations in the hybrid forms of public health professionalism were being shaped at a local level:

*there’s a variation in what they [LA public health teams] think their role is in terms of assurance verses contribution to local plans… in one local authority you hardly hear from them, they look through our outcomes and that’s that. Another local authority has dedicated health protection people in its team… they want to know exactly what’s going on all the time* [17685, PHE/NHSE, Area 2, December 2016]

The potential for local shaping was further highlighted by structural differences between LAs in Area 1:

*Some [DsPH] work at the executive level… Others may sit under the Director of Adult Social Services, some may manage their own public health budget, [for] some… it’s part of wider budget then, a pooled budget in the local authority.* [22,411, Organisational consultant, Area 1, May 2017].

This local variation was perceived to have confusing implications for public health professionalism, and whether it retained a pure form associated with medical professionalism, or whether it was more hybridised:

*I think there’s sometimes an old... the professional..., clinical badge attached to it, and this is the way we do things... And then you’ve got the more modern way of we’re a DPH, we’re on the executive board… we’re part of wider discussions around social care in local authorities… I think you’ve got that mix, and… from some areas within local authorities, there’s that lack of clarity about that role, about public health in general.* [22411, Organisational consultant, Area 1, May 2017]

Attempts to move away from the medicalisation of public health, and embrace hybridity, may therefore have varying degrees of success because of the diverse siting of DsPH within LAs. In some LAs, a newer hybridised form of public health professionalism appeared to be more clearly recognised; some public health professionals thought that the Act had helped them to instil public health principles in the development of other services within the LA:

*as a public health specialist, I’ve done some really good work in bringing adult social care specifications in line with public health needs and public health outcome framework outcomes, and I think in the past before the Health and Social Care Act, we maybe didn’t have those strong links and we didn’t have that way of thinking* [11,059, LA, Area 1, March 2016].

However, the embedding of public health throughout LA services could paradoxically dilute the perception of public health as a distinct profession:

*Public Health was a specialist field within the PCT and now it's sort of changed in that it's spread out across this Local Authority and it's supposed to be embedded into all the other departments… So it's everybody's business now whereas it used to be sort of a specialist area* [13635, LA, Area 1, June 2016]

In summary, negotiations of public health professionalism differed with local variations in structure and strategic service delivery, revealed through participants’ perceptions of the relationship between public health and other LA services, the extent to which public health can be differentiated from other services, and the extent to which it can provide leadership for service development.

### Role of Public Health England

Participants offered a range of opinions about the role of PHE, with implications about its contribution to public health professionalism. In both of our geographical areas, members of screening and immunisation teams reflected on the ambiguities arising from being PHE employees embedded within NHSE. There was uncertainty over personal responsibilities and decision-making capabilities:

*the way I see it is I work for NHS England really… But it is a bit odd because… we’re officially employees of Public Health England… there’s an issue whether I can officially speak for NHS England at a regular meeting or make decisions for NHS England, but in reality we have to, because effectively we do work for NHS England day-to-day.* [17685, PHE/NHSE, Area 2, December 2016]

There was also confusion about inter-organisation information exchange:

*frequently we will be asked for information around performance of screening programmes… there are formal routes between NHS England and PHE for those things to happen, [PHE is] not asking me, it’s asking NHS England a question, so it’s confused. And then it starts to come up with ideas about things I should be doing. Well, actually they’re not things I should be doing, it’s again what should NHS England be doing.* [18352, PHE/NHSE, Area 1, January 2017]

Ambiguities of responsibility and accountability arising from the embedding of PHE staff within NHSE thus presented challenges for PHE staff as they tried to fulfil managerial aspects of a hybrid form of public health professionalism.

The creation of PHE as a unified flagship organisation seemed to provide a positive spotlight upon the profession. However, elements associated with a ‘pure’ public health professionalism appeared somewhat inhibited by a perceived tightening of bureaucratic control associated with working for the civil service:

*in some respects it’s quite nice having Public Health England and having that focus on public health as a profession... [but] I didn’t want to become a civil servant and I still hate the civil service with a passion in certain respects for the lack of autonomy and trust… you can’t even buy a train ticket and be trusted to do it properly* [19974, PHE, Area 1, March 2017]

PHE therefore may have been seen to promote the external profile of public health, but could also be imbued strongly with personal feelings about what it means to be a civil servant: a restriction of the trust and autonomy that underpin pure forms of professionalism. There were also some concerns that PHE was staffed primarily by career civil servants who are sensitive to the principles of politics, rather than by public health specialists who embody the principles at the heart of ‘pure’ public health professionalism. The implication was that PHE was located somewhere ‘outside’ the public health community, which is completely at odds with its intended role as the profession’s flagship:

*within the public health community, there is some concern about Public Health England and its professionalism, its reporting, its accountability, its skill mix, because they don't have very many public health consultants… they're all civil servants. So things like the sugar tax report got blocked for ages. Well, if they were proper public health people, they should have been publishing that report… we're meant to stand by our principles.* [8384, LA, Area 1, November 2015]

This reference to ‘proper’ public health people implies an ‘othering’ of PHE staff, and an attempt to hold onto a ‘pure’ professional identity.

In addition to these high-level concerns about responsibilities and professional principles, our participants highlighted some more day-to-day impacts of the changes. The dividing of commissioning responsibilities between PHE and LAs may have inadvertently undermined feelings of connection with aspects of ‘pure’ public health professionalism, which makes embracing hybrid roles difficult. For example, DsPH working within LAs are no longer directly involved with screening and immunisation work, because this is overseen by PHE and NHSE. For DsPH, there was some resentment that they have lost a traditional, pure element of their professional identity:

*the local authority DsPH… have felt disenfranchised, kind of, locked out of one of the most beautiful things in public health, there was quite a lot of anger and anxiety about that loss, and it continues to roll around* [16350, Screening advisor, October 2016]

Yet for some PHE employees embedded in NHSE, who were responsible for screening, there was also some dissatisfaction with a hybrid role that seemed to foreground aspects of managerialism at the expense of advocacy working with vulnerable populations that is also perceived to be at the heart of ‘pure’ public health professionalism:

*I’m reasonably comfortable about being a PHE person, but I would also consider myself a commissioner. I don’t know if everyone else would… some of the more junior members of the team [will] say… ‘I want to be talking to local authorities about how to help vulnerable groups, we never seem to do that anymore’ … now it’s much more about, how do we performance manage this provider that’s falling apart, and [they may be] thinking ‘am I still in the right job or not?’* [17685, PHE/NHSE, Area 2, December 2016]

This shows the challenges of negotiating hybrid professionalism, and its delicate balance of principles associated with pure and controlled forms.

### Other commissioners’ perspectives upon public health

As the HSCA12 moved responsibility for commissioning the majority of public health functions away from NHS commissioners, we were interested to consider how these other commissioners perceived public health in relation to their own commissioning responsibilities. The term ‘public health’ may have had a particular interpretation by CCGs based upon their responsibilities for their local populations. There were suggestions that public health colleagues in LAs appreciated clear signs that CCGs were thinking seriously about public health:

*I have that [public health] in my title because it’s an area of interest to me. And I know public health colleagues appreciate it because they’re concerned that CCGs don’t necessarily have the public health side high up on their agenda.* [2388, CCG, Area 1, March 2015]

However, others highlighted confusion amongst CCGs about their loss of responsibility for commissioning public health services:

*Why we are the commissioners for the population, but not the public health commissioners?... if we are the commissioners, our jobs should be about how to improve it [the health of the local population] and if it is, that should include public health* [3393, CCG, Area 1, May 2015]

Around 2 years later, towards the end of our data collection period, the confusion around the location of public health commissioning was still present:

*I still don’t understand how Public Health England and public health and local authorities and where all that, sort of, you know, I still can’t quite… it’s a bit unclear and not just unclear to me, unclear to other people* [21673, CCG, Area 1, April 2017]

There were perceptions that public health might underpin commissioning in future, conducted at broader strategic levels rather than at localised CCG levels, but which seemed to emphasise managerial aspects of outcomes and contract management (‘controlled professionalism’) rather than aspects associated with more pure forms of public health professionalism:

*I think you're going to find you don't really need commissioners in the numbers you've got now. I think you’ll need some very good strategic commissioners who work across that population base, probably a bit more public health then anything. Some good outcome setting and some good contract management and then you leave the accountable care organisation to get on with it don't you?* [7541, Commissioning Support Unit, Area 1, October 2015]

In summary, there appeared to be some enduring confusion amongst other commissioners about the remit of CCGs, PHE and LAs in relation to public health. This confusion seemed to arise from a conflation of ‘public’ and ‘population’ health, and the localism agendas inherent to the HSCA12 reforms. There were suggestions that controlled forms of public health professionalism might underpin future commissioning at a broad strategic level.

## Discussion

The WHO Regional Office report cited earlier [3] highlights significant problems affecting public health, and offers a wide-ranging template of work to be done to mitigate these. However, the very breadth of proffered solutions raises issues: what is the distinctive contribution of the public health profession? Our paper has explored the role of public health following significant reforms to the commissioning system in England. The key public health elements of these reforms – transfer of public health responsibilities and professionals to local authorities, and creation of a new national public health body – provide a lens through which to explore more general issues about the nature of public health professionalism, which may be of relevance more widely as health authorities and governments seek to engage with the WHO public health agenda.

One of our participants spoke to the increasingly-wide recognition that public health is now ‘everybody’s business’. For this to be meaningful it requires recognition that stakeholders situated at different locations in a health and care system have different responsibilities for public health [43]. Historical analysis suggests that there is no optimal position within a system to site the public health profession. Therefore, where public health professionals sit in a system in absolute terms is less important than their ability to develop relationships, negotiate their roles, and provide expert influence across that system. To do this, a strong professional identity is important, and our data show that public health professionals are continuing to negotiate ‘hybrid’ or ‘organising’ forms of professionalism [17] as they attempt to assert their identity within the post-HSCA12 system. Our data show the potential for hybrid forms of public health professionalism to be shaped at a local level according to local variations. DsPH who are able to negotiate the political imperatives of local government are potentially able to thrive, yet their wings may be clipped by their position within their organisation relative to other senior leaders. This illustrates how professionals have to reconcile the competing ideologies and interests of their own profession and those of the organisation(s) in which they are working [19].

One of the main challenges in this reconciliation for public health professionals appears to come when trying to realise *values* in and around their work. The core quality that they are not prepared compromise on in their hybrid or organising roles seems to be the advocacy perceived as intrinsic to public health. Our data reveal difficulties for some public health professionals working within PHE who are bound by the civil service code of conduct, and challenges for those working in LAs in advocating for services threatened by financial or political considerations. The House of Commons report into public health post-HSCA12 suggests that whilst the move of public health to local government has theoretically increased the democratic narrative around public health, it is not clear how genuine the idea of a democratic accountability is in a time of austerity which sees public services being cut [37].

One of our participants suggested that public health principles may inform future strategic commissioning, which tallies with calls for a new paradigm of health services reform that uses the skills and principles of public health [44]. At the time of writing, English health policy is increasingly looking to integrated ‘place-based’ commissioning, with the country divided into 44 different health economies in which NHS organisations, in partnership with local councils and others, will take collective responsibility for managing resources, delivering NHS standards, and improving the health of the population they serve [45]. There is therefore an opportunity to place public health at the fulcrum of these ‘local’ health and care economies, because this ‘place-based’ approach is intended to achieve a breakdown of organisational silos and increase collaboration between stakeholders. However, the central role for public health professionals remains unrealised. These local health economies are works in progress, generally lacking in clarity and detail around financial planning, workforce implications, and implementation plans [45, 46]. Some public health leaders have argued that a rhetoric of prevention is undermined by a reality of austerity, and development has thus far lacked public health input [43, 46]. There are concerns that expectations are too high, and that public health professionals simply will not be able to deliver what they are expected to do [43].

The WHO agenda suggests there may be an important role for a national public health body to set an agenda and provide support to public health professionals in achieving comfortable and sustainable hybrid professionalism. In England, this body is PHE, but our data show that its role in supporting public health professionalism appears unclear. In particular, there is uncertainty for some public health professionals within PHE about their roles and professional identities, who may struggle with taking on roles involving commissioning and provider-management, and who may dislike being members of the civil service. The root of this complexity may be found in Horton’s analysis of the changing relationship between the ‘public service ethos’ and the civil service, which argued that the civil service has become increasingly managerial and commercial, performing a primarily administrative role rather than an advisory one [47]. Ten years after Horton’s analysis, our data suggest that PHE foregrounds continuing discrepancies and divergences between the ‘service ethos’ inherent to purer forms of public health professionalism, and the more managerial civil servant role that it may now represent.

Of relevance to all of these challenges is the conceptual confusion between public health and population health. This confusion is writ large in the WHO list of ‘essential public health operations’, which is so broad that no one professional group alone could possibly address it. In their proposals for how the public health profession may strengthen global health systems, Bloland et al. [5] define public health as ‘prevention-oriented population health’. This definition renders public health synonymous with prevention of disease. However, population health can also be defined as a focus on health outcomes of a group of individuals, and patterns of health determinants, linked by specific policies and interventions: it is the ‘bigger picture’, and public health as a profession does not have control over major determinants of health such as deprivation, education and income [48]. There remains a need to clarify the role(s) and expectations for public health as a specialist profession in helping to fulfil broader population health goals that go beyond curtailing outbreaks of infectious diseases.

### Reflections

The work presented in this paper benefits from being part of a longitudinal study with the resources to collect data from a large number of senior stakeholders, and the capacity to keep abreast of contemporaneous post-HSCA12 policy development. Consideration of the impact of the HSCA12 upon public health professionalism was not imposed by the researchers upon the participants; it emerged during exploration of our main research questions, in particular (but not solely) arising from our analyses of sexual health and screening programmes. Our reanalysis to explore this emic phenomenon benefits from the use of theoretical concepts of professionalism as an analytic lens. We recognise that our work is taken from a subset of participants in two areas of England, and that a broader range of participants would be beneficial. Future research could usefully explore these issues with a broader range of public health professionals, including those working in service delivery roles. We present this paper as a contribution to a much wider conversation about the development of public health professionalism.

## Conclusions

In this paper we have used the most recent major reforms to the English National Health Service and local government, the Health and Social Care Act 2012, as a lens through which to explore the changing nature of public health professionalism. Where public health professionals sit in a health system in absolute terms is less important than their ability to develop relationships, negotiate their roles, and provide expert public health influence across that system. A conflation between ‘population health’ and ‘public health’ fosters unrealistic expectations of the profession. Public health may be best placed to provide leadership for other stakeholders and professional groups working towards improving health outcomes of their defined populations, but there remains a need to clarify the role(s) that public health as a specialist profession has to play in helping to fulfil broader population health goals.

## Abbreviations

CCG: Clinical Commissioning Group
DPH: Director of Public Health
HSCA12: Health and Social Care Act 2012
LA: Local Authority
MOH: Medical Officer of Health
NHS: National Health Service
NHSE: NHS England
PCT: Primary Care Trust
PHE: Public Health England
WHO: World Health Organization
